# Exact and Metaheuristic Approaches for a Bi-Objective School Bus Scheduling Problem

**DOI:** 10.1371/journal.pone.0132600

**Published:** 2015-07-15

**Authors:** Xiaopan Chen, Yunfeng Kong, Lanxue Dang, Yane Hou, Xinyue Ye

**Affiliations:** 1 Key Laboratory of Geospatial Technology for the Middle and Lower Yellow River Regions, Ministry of Education, Henan University, Kaifeng, Henan 475004, China; 2 College of Computer and Information Engineering, Henan University, Kaifeng, Henan 475004, China; 3 Department of Geography, Kent State University, Kent, OH 44242, United States of America; Bangladesh University of Engineering and Technology, BANGLADESH

## Abstract

As a class of hard combinatorial optimization problems, the school bus routing problem has received considerable attention in the last decades. For a multi-school system, given the bus trips for each school, the school bus scheduling problem aims at optimizing bus schedules to serve all the trips within the school time windows. In this paper, we propose two approaches for solving the bi-objective school bus scheduling problem: an exact method of mixed integer programming (MIP) and a metaheuristic method which combines simulated annealing with local search. We develop MIP formulations for homogenous and heterogeneous fleet problems respectively and solve the models by MIP solver CPLEX. The bus type-based formulation for heterogeneous fleet problem reduces the model complexity in terms of the number of decision variables and constraints. The metaheuristic method is a two-stage framework for minimizing the number of buses to be used as well as the total travel distance of buses. We evaluate the proposed MIP and the metaheuristic method on two benchmark datasets, showing that on both instances, our metaheuristic method significantly outperforms the respective state-of-the-art methods.

## Introduction

Due to the large scale of school merge and closures in China since the late 1990s, more and more primary and secondary schools have begun providing school bus service. According to the authors’ survey, in 2013, there are already thousands of school buses in service in cities such as Shanghai, Guangzhou, Shenzhen, Changchun, Xiamen and Qingdao. However, most of the school buses are operated by schools respectively. Recognizing the importance of school bus safety and operation efficiency, some local authorities, such as Binzhou, Chifeng, Wuli, Youxian and Zhuzhou, have proposed to provide regional level bus service for all schools. The Ministry of Education of China has launched a statewide school bus pilot program in six counties in 2011, aiming to explore the best practice of school bus operations in China. For local government and transportation company, school bus route planning is already a difficult task; still, it is crucial to serve with quality, equality and efficiency.

The school bus routing problem (SBRP) is concerned with how to transport students to and from schools in the safest, the most economical and convenient manner [1]. SBRP research has shown that the cost of school bus service can be reduced greatly by optimizing the school bus routes while satisfying service quality at the same time. SBRP consists of five sub-problems[2]: data preparation, bus stop selection, bus route generation, school bell time adjustment, and route scheduling. The bus route generation problem is to construct bus trips for each school. The route scheduling is also called bus scheduling wherein trips for each school are given. By bus scheduling, many sequences of bus trips are constructed and make each trip be finished within the time windows of designated school. The bus utilization can be improved by school bus routing and scheduling. Based on the bus service designed for a single-school or a multi-school system, SBRP can be classified into two general categories: single-school problem and multi-school problem[2].

Most existing SBRP researches focus on the single-school routing problem. Considering the limited capacity of each bus, the bus routing problem is usually modeled as the capacitated vehicle routing problem (CVRP). In a CVRP, a fleet of vehicles (the buses) serve a set of clients (the bus stops). The objective is to minimize the total travel distance and/or the number of buses required. For satisfying school bus service regulations, various constraints must be satisfied: bus capacity, student’s maximum riding time in a bus, and time windows of schools. Such problems are usually solved by exact methods [3–7] or (meta)heuristics such as ant colony optimization (ACO)[8, 9], time savings heuristic and sweep method [10], genetic algorithm (GA) [11–13], and tabu search [14–16]. Since CVRP is a NP-hard problem in strong sense, only the small-size problems can be solved by exact methods such as branch-and-cut and column generation[17, 18].

The multi-school SBRP is a special case of the pickup and delivery problem with time windows (PDPTW) [19]. In a PDPTW, goods or passengers (students) have to be transported from different origins (bus stops) to different destinations (schools). PDPTW is more difficult than CVRP [17]. Braca et al. developed a randomized location-based heuristic (RLBH) to construct bus routes by inserting a pair of bus stop and school into route solution sequentially [20]. Dang et al. proposed a PDPTW-based neighborhood search algorithm that is able to find much better solution for SBRP, though it would require redundant computation time[21]. Campbell et al. developed a three-phase heuristic algorithm for multi-school SBRP[22]. Since the size of multi-school SBRP is usually large in terms of the number of students, the number of bus stops and the size of bus fleet, it is difficult to be solved efficiently using existing PDPTW algorithms. Therefore, in most of the existing literature, the multi-school SBRP is separated into two sub-problems: the single-school routing problem and bus scheduling problem[19, 23–27]. When the bus trips for each single school are given, the bus scheduling problem is usually optimized by modeling it as a VRP with time windows (VRPTW)[25].

This paper proposes two approaches for solving the bi-objective school bus scheduling problem: an exact method of mixed integer programming (MIP) and a metaheuristic method. Both the homogeneous and heterogeneous fleet of buses are considered in this research. We rewrite the MIP formulations for bi-objective bus scheduling problem and solve it using the MIP optimizer IBM ILOG CPLEX. We also propose a simulated annealing algorithm framework to minimize the number of buses and the total travel distance. The performance of the proposed models and algorithms is tested on two sets of benchmark instances.

This paper is organized as follows. The next section defines the school bus scheduling problem. A bus type-based formulation for heterogeneous fleet school bus scheduling problem is introduced in this section. Section 3 proposes a generic simulated annealing framework. Based on the framework, three algorithms are implemented to solve single-school SBRP, homogeneous fleet school bus scheduling problem and heterogeneous fleet school bus scheduling problem respectively. Section 4 shows the computation results for benchmark instances. The optimization performance of exact method and metaheuristic algorithm are compared in detail. The last section offers the research findings and conclusions.

## Problem Definition and Modeling

### 2.1 Problem definition

In SBRP, a number of prerequisites, such as a set of schools, a set of student stops, a set of depots, a fleet of school buses and their geographic background, must be available for school bus route planning. The SBRP dataset should include both geographic and attribute information. The bus travel distances and times between schools, stops and depots can be prepared by geospatial analysis of the transportation network in GIS. The objective of school bus route planning is to minimize the number of school buses and the total bus travel distance while satisfying service qualities such as the student maximum riding time in a bus, the earliest and latest arrival times to their schools, and the maximum bus capacity.

**Fig 1:**
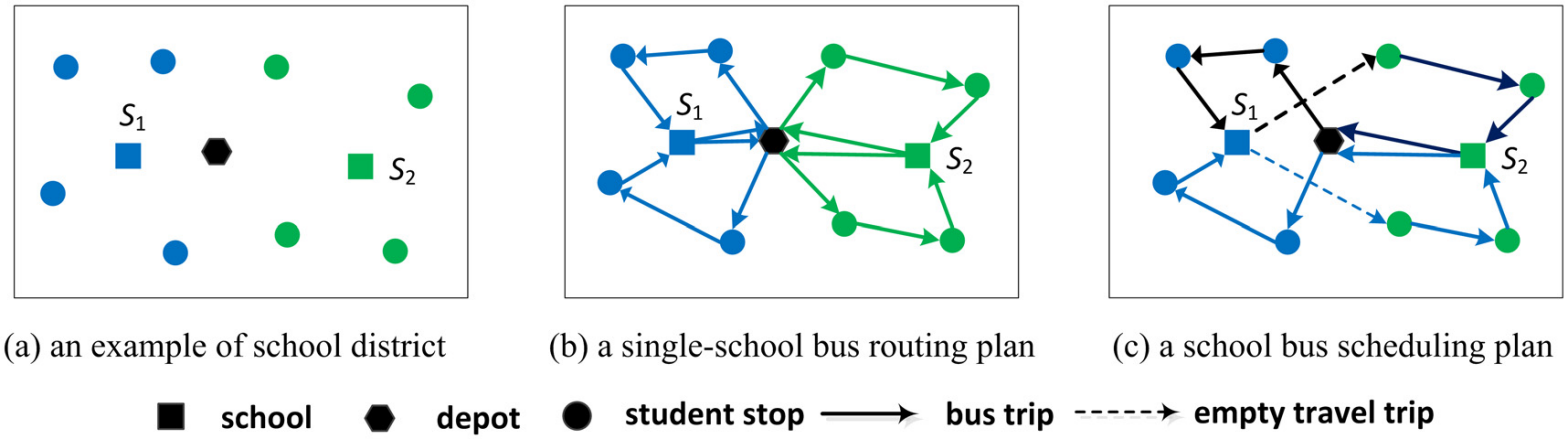
Two bus route plans for an example of school district.

Fig 1 illustrates two bus route plans for an example of school district. There are 2 schools and 8 student stops in the school district (Fig 1a). The student stops are filled with the same color as their schools. For school bus service in this district, Fig 1b shows a single-school bus route plan, in which there are two trips for school *s*_1_ and *s*_2_ respectively. Each trip consists of the depot, a sequence of bus stops and their destination school. One bus starts from the depot, picks up students of each bus stop in its trip, delivers them to their school, and then returns to the depot. When two trips can be served by the same bus in sequence without violating service constraints, they are assigned to the same bus (Fig 1c). This is a school bus scheduling problem, which may reduce the number of buses required. In Fig 1c, the dotted line represents the empty travel trip between two connected trips.

This paper focuses on the bus scheduling problem. In following sections, the MIP models for school bus scheduling problems are formulated in detail. Note that the bus trip in this paper refers to a bus route for a single school. The bus scheduling is to assign buses to trips, thus one bus can serve one or more bus trips.

### 2.2 MIP formulation for homogeneous fleet school bus scheduling

If the bus trips for each school are given, the bus scheduling problem is to assign buses to trips, and find the optimal solution with minimum number of buses and minimum bus travel distance. Let *V*’ be the set of single-school bus trips, and the number of trips is *n*. Each rip is composed of a sequence of bus stops and their designation school. The travel distance,*d*_*ij*_, from trip *i* to trip *j*, can be determined by the travel time from the designated school of trip *i*, to the first stop of trip *j*. Set *S* is a set of schools (|*s*| = *m*). Each school *s* (*s* ∈ *S*) has a fixed time windows [*e*(*s*), *l*(*s*)]. The students should arrive to the school *s* within the associated time window. Table 1 shows the parameters and decision variables for bus scheduling with a homogeneous fleet of buses.

**Table 1:** Parameters and decision variables for homogeneous fleet bus scheduling problem.

Parameters	
*S*	The set of schools
*e*(*s*),*l*(*s*)	The time windows for school *s*, *e*(*s*) ≤ *l*(*s*), *s* ∈ *S*
*V*’	The set of single-school bus trips
*V*	The set of bus trips and the bus depot 0, *V* = *V*’ ⋃ 0
*s*(*i*)	The destination school of bus trip *i*, *I* ∈ *V*’, *s*(*i*) ∈ *S*
*p_i_*	The service time for bus trip *i*, including bus travel time, pickup and delivery time, let *p*_0_ = 0, *i* ∈ *V*
*d_ij_*	The distance from *i* to *j*, *i*, *j* ∈ *V* |*i* ≠ *j*
*t_ij_*	The travel time from *i* to *j*, *i*, *j* ∈ *V* |*i* ≠ *j*
*e*_0_,*l*_0_	The earliest and latest departure time for buses from the depot
Decision variables	
*x_ij_*	If a bus serves trip *i* and leaves to serve trip *j* immediately, *x_ij_* = 1; otherwise *x_ij_* = 0, *i*, *j* ∈ *V* |*i* ≠ *j*
*y_i_*	The arrival time at the destination school of trip *i*, *i* ∈ *V*’, let *y*_0_ = 0

The bi-objective MIP formulation for homogeneous fleet school bus scheduling problem (MIPHO-B) is shown as follows:

$$\text{Minimize}:M_{0}\sum_{i\in V^{\prime }}x_{0i}+\sum_{i\in V^{\prime }}\sum_{j\in V^{\prime },i\neq j}d_{ij}x_{ij}+\sum_{i\in V^{\prime }}x_{0i}d_{0i}+\sum_{j\in V^{\prime }}x_{j0}d_{j0}$$ (1)

$$\text{Subject to}:\sum_{i\in V}x_{ij}=1\;,\forall j\in V^{\prime }$$ (2)

$$\sum_{j\in V}x_{ij}=1\;,\forall i\in V^{\prime }$$ (3)

$$e(s(i))\leq y_{i}\leq l(s(i)),\forall i\in V^{\prime }$$ (4)

$$y_{i}+t_{ij}+p_{j}-y_{j}\leq (1-x_{ij})M,\forall i\in V,j\in V^{\prime }|i\neq j$$ (5)

$$x_{ij}\in \{0,1\},\;\forall i,j\in V^{\prime }|i\neq j$$ (6)

$$y_{i}\in \{0,1,2,\ldots \},\;\forall i\in V^{\prime }$$ (7)

The objective Eq (1) is to minimize the number of buses and total travel distance. The first part of Eq (1) denotes the number of buses. The parameter *M*_0_ is a big number that ensures the number of buses is as the primary objective and minimizes the two objectives in a lexicographic manner. Eqs (2) and (3) ensure that each trip is served by exactly one bus, and the bus must leave to another trip or the depot. Eq (4) assure that the bus serving trip *i* should arrive to school *s*(*i*) at any time within the school time window. Eq (5) state the accumulation of bus travel time:(*y*_*i*_ + *p*_*j*_ + *t*_*ij*_−*y*_*j*_)*x*_*ij*_ = 0. If the arc (*i*,*j*) is traversed by a bus (*x*_*ij*_ = 1), then *y*_*i*_+*p*_*j*_+*t*_*ij*_ = *y*_*j*_. The non-linear constraints can be transformed to linear inequalities Eq (5) by using the big *M* parameter. Eqs (6) and (7) are the constraints on decision variables.

### 2.3 MIP formulation for heterogeneous fleet school bus scheduling

Unlike the assumption of a set of identical buses in the homogenous fleet model, a fleet of buses usually have different capacities and purchase cost in the heterogeneous fleet problem. There are two approaches of defining the three-index decision variables *x*_*ijk*_ in problem formulation, where *k* presents a vehicle[17, 28] or a type of vehicles[29]. Kim et al. developed a bus-based MIP model (MIPHE) for heterogeneous fleet school bus scheduling problem[25], and solved a set of problem instances by CPLEX. We rewrite the model by changing the bus-based decision variables to the bus type-based variables, aiming to reduce the model complexity. In the bus-based formulation, *k* in the decision variables *x*_*ijk*_ denotes the bus *k* to serve arc (*i*,*j*) in which *i* and *j* denotes the single bus trip. But, in the bus type-based formulation, the *k* denotes the bus of type *k* to serve arc (*i*,*j*). In addition, the fixed cost for each type of buses is also introduced in our objective function, which is different from that defined in MIPHE. Most parameters of the formulation are the same as the homogeneous problem defined in Table 1; additional parameters and decision variables are shown in Table 2.

**Table 2:** Parameters and decision variables for heterogeneous fleet school bus scheduling problem.

Parameters	
*K*	The set of bus types
*F_k_*	The fixed cost of a bus of type *k*, *k* ∈ *K*
*Q_k_*	The capacity of a bus of type *k*, *k* ∈ *K*
*m_k_*	The number of buses of type *k*, *k* ∈ *K*
*c_i_*	The number of students serviced by trip *i*(*i* ∈ *V*), let *c*_0_ = 0
*E_ik_*	If a bus of type *k* can serve route *i*(*Q_k_* ≥ *c_i_*),*E_ik_* = 1; otherwise, *E_ik_* = 0. *i* ∈ *V*’, *k* ∈ *K*
Decision variables	
*x_ijk_*	If a bus of type *k* serves trip *i* and leaves to serve trip *j* immediately, *x_ijk_* = 1; therwise *x_ijk_* = 0.*i*, *j* ∈ *V* |*i* ≠ *j*, *k* ∈ *K*
*y_ik_*	The arrival time at the destination school of trip *i* by a bus of type *k*, *i* ∈ *V*’, *k* ∈ *K*

The bi-objective MIP formulation for heterogeneous fleet school bus scheduling problem (MIPHE-B) is shown as follows:

$$\text{Minimize:}\sum_{k\in K}F_{k}\sum_{j\in V^{\prime }}x_{0jk}+\sum_{k\in K}\sum_{i\in V^{\prime }}\sum_{j\in V^{\prime },i\neq j}d_{ij}x_{ijk}+\sum_{i\in V^{\prime }}x_{0ik}d_{0i}+\sum_{j\in V^{\prime }}x_{j0k}d_{j0}$$ (8)

$$\text{Subject to:}\sum_{k\in K}\sum_{i\in V}x_{ijk}=1\;,\forall i,j\in V^{\prime }|j\neq i$$ (9)

$$\sum_{i\in V}x_{iuk}-\sum_{j\in V}x_{ujk}=0,\forall u\in V^{\prime },k\in K$$ (10)

$$\sum_{i\in V}x_{0ik}\leq m_{k},\forall k\in K$$ (11)

$$c_{i}x_{ijk}\leq Q_{k}x_{ijk}\;,\forall i\in V\prime ,j\in V|j\neq i,k\in K$$ (12)

$$c_{j}x_{ijk}\leq Q_{k}x_{ijk}\;,\forall j\in V\prime ,i\in V|j\neq i,k\in K$$ (13)

$$e(s(i))\leq y_{ik}\leq l(s(i)),\forall i\in V^{\prime }$$ (14)

$$y_{ik}+t_{ij}+p_{j}-y_{jk}\leq (1-x_{ijk})M,\forall i\in V^{\prime },j\in V^{\prime },k\in K$$ (15)

$$x_{ijk}\in \{0,1\},\;\forall i,j\in V|i\neq j,k\in K$$ (16)

$$y_{ik}\in \{0,1,2,\ldots \},\;\forall i\in V^{\prime },k\in K$$ (17)

The objective Eq (8) is to minimize the total fixed cost of buses and total travel distance. Eqs (9) and (10) ensure that each trip is served by only one bus, and the bus shall then leave to another trip or the depot. Eq (11) ensure that the number of used buses of type *k* does not exceed the total number of type *k* buses. Eqs (12) and (13) assure that a bus of type *k* must have enough capacity to serve a trip. Eq (14) assure that the bus of type *k* serving route *i* should arrive to school *s*(*i*) at any time within the school time window. Eq (15) state the accumulation of bus travel time: (*y*_*ik*_+*p*_*j*_+*t*_*ij*_–*y*_*ik*_)*x*_*ijk*_ = 0. Eqs (16) and (17) are the constraints on decision variables.

The bus type-based formulation for the school bus scheduling problem can reduce the model complexity significantly. Comparing to the bus based model MIPHE, the number of decision variables is reduced from (*n* ^2^+*n*)*m* to (*n* ^2^+*n*)*k*, where *n*, *m* and *k* represent the number of bus stops, the number of buses and the number of bus types. For an example of problem instance with 50 bus stops and a fleet of 20 buses, we need to define 51,000 decision variables in the bus-based formulation. If the buses are categorized into 3 types, we need to define 7,650 decision variables in bus type-based formulation, where 85% of decision variables are reduced. The parameters *F*_*k*_ in the objective function encourage the model solver to select small buses with smaller fixed costs. In model building for a problem instance, the model can be simplified by introducing parameters *E*_*ik*_. If *E*_*ik*_ *E*_*jk*_ = 0 (or *E*_*ik*_ = 0), the decision variable *x*_*ijk*_ = 0 (or *y*_*ik*_ = 0) can be deleted from the model, and the related elements in the objective function and constraints can also be deleted. In fact, all the Eqs (12) and (13) can be removed from the model. In addition, if *e*(*s*(*i*))+*t*_*ij*_+*p*_*j*_>*l*(*s*(*j*)), the connection between trips *i* and *j* is infeasible. In such a case, the decision variable *x*_*ijk*_ = 0; thus the related elements in the objective function and constraints can also be deleted, and some Eq (15) will also be deleted.

Since the difference of capacity and cost between a fleet of buses is considered in the heterogeneous fleet problem, it is more complex than the homogenous fleet problem. Technically, the MIP model for the homogeneous fleet problem is a special case of the heterogeneous fleet problem, where all the buses are assumed to be identical. In addition. Compared with the bus-based model MIPHE, the bus type-based model MIPHE-B reduces the number of decision variables significantly; thus, its computational complexity can be reduced.

## Metaheuristic Algorithm

### 3.1 Algorithm framework

In this paper, the multi-school SBRP is decomposed into single school bus routing problem and school bus scheduling problem. The former problem is equivalent to open CVRP with a constraint of maximum riding time of students. The latter can be converted to VRPTW by treating each trip as a virtual bus stop [25]. Based on the neighborhood search operators for VRP, we propose a two-stage algorithm framework for both problems in the following steps:

1. DataInput();
2. BuildInitialSolution();
3. RouteMinimize_ILS_SA();
4. LengthMinimize_ILS_SA();
5. OutputSolution();

Step 1 reads the data of a SBRP instance. Step 2 generates a set of feasible routes as the initial solution. The feasible routes must meet specific constraints. Step 3 is to minimize the number of routes using neighborhood operators coupled with simulated annealing. Since the multi-objective function in step 3 encourages reducing the number of bus routes, additional step 4 is used to minimize the total travel distance. The output solution in step 3 is fed as the initial solution of step 4.

There are two general objectives for SBRP in this research: minimizing the number of buses and minimizing the total travel distance. Since the capital cost per bus is significantly higher than the travel cost per distance unit [30], the number of routes is of higher priority than the travel distance. However, it is very difficult to obtain a solution that all the objectives are optimal simultaneously. The objective function of total travel distance commonly used in VRP may lead the search to the solutions with a small travel distance and makes it impossible to remove routes[31]. Therefore, we use a lexicographic multi-objective function to evaluate the neighbourhood solutions in step 3:

$$\text{Cost}(S)=\alpha |R|-\beta {\sum_{r\in R}\left|r\right|}^{2}+\gamma \text{Dist(}R)$$ (18)

In the function, the |*R*|, Dist(*R*)and |*r*|^2^ are the number of routes, the total length of all routes, and the sum of squared route stop numbers respectively. Given the parameters *α* ≫ *β* ≫ *γ*, the neighbourhood moves prefer to delete some routes if possible. In step 4, only the function of Dist(*R*) is used to minimize the total travel distance.

After the construction of an initial route solution by a simple heuristic algorithm, the key steps of this algorithm are to improve the solution by exploring the neighborhood solutions. Local search is a metaheuristic method for solving combinatorial optimization. Various local search algorithms iteratively improves the solution of a problem by applying local changes to current solution until optimal solution is deemed to be found or a stop criterion is met. In the process of searching a better solution, once a feasible neighborhood solution is identified and its objective is better than the current solution, the current solution will be replaced by the new one. Local search has proven to be an effective technique for generating good solutions to instances of the classical VRP as well as variants such as VRPTW[32].

A general local search operator exchanges μ nodes from one route with λ nodes from another. The local search operators such as one-point move, two-point move and 2-opt move can work on a single route or different routes, while cross-exchange is an inter-route operators that only work on two routes simultaneously[33]. One-point move attempts to relocate an existing node to a new position, which can reduce the number of routes and create more opportunities for other operators. Two-point move swaps two nodes to reduce route distance. 2-opt move tries to improve a solution by removing two edges from the solution and replace them with two new edges. Cross-exchange move tries to remove two edges respectively from two different routes and replace them with four new edges. The repeat of the four local search operators will improve the route solution gradually.

The local search algorithm has drawbacks: the quality of solution is closely related with the initial solution and the spatiotemporal structure of the problem instance. Meanwhile, the procedure is very easily trapped into local optima. In general, such a local optimum is not a global optimum[34]. Even worse, there is usually no guarantee that the value of the objective function at an arbitrary local optimum is close to the optimal value. Some methods have been proposed to solve the problem of local optimum, such as simulated annealing and tabu search. In this paper, the technique of simulated annealing[35] is combined with local search operators. In the local search operations, we accept some solutions that make route length longer by simulated annealing, but may escape from local optima and realize the search in depth and diversification. The general local search procedure coupled with simulated annealing is designed in the following steps:

### Algorithm 1: The local search with simulated annealing

**Input**: the SBRP instance (*S*), the route solution (*sol*), the initial temperature (*t*_0_), the cooling rate (*α*), the acceptance criterion for neighborhood solutions (*rule*), and the maximum loops of local search (*maxloop*)

1. *t*←*t*_0_;
2. *sol*_*best*_←*sol*;
3. for (*i*: = 1; *i*≤*maxloop*; *i*++) {
4. *perm* = *NodePermutation*(*S*)
5. *OPM* (*S*, *perm*, *t*, *rule*);
6. *TPM* (*S*, *perm*, *t*, *rule*);
7. *TOM* (*S*, *perm*, *t*, *rule*);
8. *CE* (*S*, *t*, *rule*);
9. *t*←*α*×*t*;
10. }

**Output**: the best solution.

This procedure can be implemented for school bus routing and bus scheduling for the purpose of minimizing the number of routes and minimizing the total travel distance. Line 4 permutes the nodes randomly, which will change the node searching sequence in each iteration for solution diversification. From line 5 to line 8 in the loop, one-point move search, two-point move search, 2-opt move search, and cross-exchange search shall be executed sequentially. In each local search function, according to feasibility check, objective evaluation and acceptance criterion; the neighborhood solution will either be accepted as the current best solution so far or rejected. In line (9) the temperature drops, and the possibility of accepting a new solution with longer travel distance in next loops will be decreased gradually.

### 3.2 Local search operators

The route solution *sol* is gradually improved by four local search operators: one-point move, two-point move, 2-opt move and cross-exchange move. Algorithm 2 shows the details of the one-point move search. The search procedures for other three operators are similar to one-point move search.

### Algorithm 2: *OPM* (*S*, *perm*, *rule*)

**Input**: the SBRP instance (*S*), the sequential or randomized node list (*perm*), and the criteria to accept a new solution (*rule*). Note that the instance *S* includes the current best route solution (*sol*_*best*_), the current temperature (*t*) and other information

1. FOR EACH (*p* in *perm*){
2. *Nlist* ←OPM_neighborhood(*p*, *S*);
3. FOR EACH (p* in *Nlist*) {
4. IF (check_feasiblity(*p*, p*) = = false) CONTINUE;
5. sol*←OPM_ neighborhood solution(*p*, p*);
6. IF (is_better(*sol*_*best*_, sol*) = = false) CONTINUE;
7. IF (*rule* = = FA) {*sol*_*best*_←sol*; BREAK;}
8. IF (*rule* = = BA) {
9. IF (is_better2(sol*_best_, sol*) = = false) CONTINUE;
10. sol*_best_←sol*;
11. }
12. IF (*rule* = = BA) *sol*_*best*_←sol*_best_
13. }

**Output**: the current best solution (*sol*_*best*_)

For each node *p*, the algorithm constructs its neighborhood list (*Nlist*) at first (line 2). For each neighbor node p* in the neighborhood list, if the node *p* can be inserted after node p* without violation of the constraints, the objective function for this feasible move will be evaluated. Otherwise, the operator will try another neighbor position (line 4). If the neighborhood solution (line 5) is better than the current best solution *sol*_*best*_ or, at least acceptable with the simulated annealing criteria (line 6), the best solution will be updated according to acceptance criterion (*rule*): first acceptance (FA) or best acceptance (BA) (line 7–12). The function is_better(*sol*_*best*_, sol*) evaluates the solution quality of sol* compared with that current best solution *sol*_*best*_; The function is_better2(sol*_best_, sol*) is used to select the best neighborhood solution. For minimizing the number of routes, the three elements in objective Eq (18) are in a lexicographic order.

### 3.3 Solvers for school bus routing and scheduling

Based on the algorithm framework and local search operators discussed in Section 3.1 and 3.2, we developed three solvers for single-school SBRP, homogeneous fleet school bus scheduling problem and heterogeneous fleet school bus scheduling problem respectively. The solvers are used to plan bus routes. Firstly, the bus trips for each school in a multi-school system is generated by the single-school solver. Secondly, the buses are assigned to the trips by using the homogeneous fleet or heterogeneous fleet school bus scheduling solver. The output data from the first solver is fed into the second solver. In each solver, a simple savings-based[36] heuristic algorithm is used to construct an initial solution. A two-stage metaheuristic algorithm is then used to improve the solution. The first stage minimizes the number of routes, which reduces the total number of buses needed for students. The second stage optimizes the total bus travel distance, which will reduce the total bus travel cost. The procedures for the two stages are designed in the same manner, except for the evaluation of solution objectives.

The VRP with a heterogeneous fleet is considered much harder than corresponding problems with a homogeneous fleet[37]. The heterogeneous fleet school bus scheduling problem is also much more difficult to solve than the homogeneous fleet problem. For initial solution construction, we divide the heterogeneous fleet problem into multiple homogeneous fleet sub-problems according to the types of buses. First, the trips and the types of buses are sorted in an ascending order. Second, the trips are divided into multiple groups according to the capacities of buses; a type of buses with minimum capacity is assigned to each group of trips as well. Third, the saving-based heuristic algorithm is used to construct the route solution for each group of trips. In case the number of one type of buses is not enough to serve the sub-problem, the types of buses for all routes need to be adjusted appropriately. Fourth, the route solutions of each group are merged into an initial route solution for the whole heterogeneous fleet problem. The initial solution is then improved by the two-stage algorithm. Comparing with the neighborhood point moves in solving homogeneous fleet problem, it is needed to check the bus types for any possible point moves.

The solver programs are implemented by C++ programming and compiled as 32-bit command-line executables with parameter setting options. The parameters includes the maximum number of loops (*maxloop*), the initial temperature for simulated annealing(*t*_0_), the cooling rate of temperature(*α*), the length of neighborhood list (*NList*), the acceptance criteria for solution updating (*rule*), and the randomized node search strategy (*random*).

## Computational Results

### 4.1. Results for HOMO and HETERO instances

The HOMO and HETERO benchmark instances are collected from literature [25]. There are 15 instances for homogeneous fleet problem and heterogeneous fleet problem respectively. Table 3 shows the size of all instances. For each instance, the school data includes the school ID, the spatial coordinates *x* and *y* and the school time window. The bus trips for each school are given, which include the trip ID, the coordinates of the first stop in the trip, destination school ID, and the bus service time. The bus data for heterogeneous fleet instances include the bus ID and the bus capacity.

**Table 3:** The school bus scheduling instances HOMO and HETERO.

Homogeneous fleet instances					Heterogeneous fleet instances				
Instance	#schools	#trips	#students	#buses	Instance	#schools	#trips	#students	#buses
homo1	1	3	143	3	hetero1	1	7	335	7
homo2	2	13	611	13	hetero2	2	13	596	13
homo3	3	20	975	20	hetero3	3	21	1012	21
homo4	4	23	1154	23	hetero4	4	24	1199	24
homo5	5	29	1448	29	hetero5	5	32	1610	32
homo10	10	49	2405	49	hetero10	10	58	3002	58
homo20	20	106	5097	106	hetero20	20	120	6012	120
homo30	30	161	8155	161	hetero30	30	187	9415	187
homo40	40	234	11528	234	hetero40	40	239	12136	239
homo50	50	309	15238	309	hetero50	50	286	14352	286
homo60	60	372	18551	372	hetero60	60	345	17350	345
homo70	70	430	21554	430	hetero70	70	408	20723	408
homo80	80	461	23066	461	hetero80	80	469	23686	469
homo90	90	514	25736	514	hetero90	90	514	25736	514
homo100	100	562	28175	562	hetero100	100	562	28175	562

We build models for all the instances according to the MIP formulations proposed in Section 2.2 and 2.3. The model parameters are the same as literature [25]: the travel distance *d*_*ij*_ is an integer number calculated by Euclidean distance with a truncated fraction; the bus speed is assumed to be 1 distance unit per time unit, and thus *t*_*ij*_ = *d*_*ij*_. In the objective function, the travel distance from the bus depot to the first trip and from the last trip to the depot are not considered in the total distance travelled by all the buses. In the model MIPHE-B, the bus type data are counted from the bus data. There are three types of buses in the heterogeneous fleet instances; and the fixed costs for each type of buses are assumed to be 100000, 90000 and 80000 for buses with capacity of 70, 50 and 40 respectively. The parameters *M* and *M*_0_ are set to 10^9^ and 10^5^ respectively.

All the models are solved by the MIP solver IBM ILOG CPLEX 12.6 (64bits). CPLEX is installed on a personal computer with the following hardware configuration: Intel(R) Core2 Duo E7600 3.06G CPU, 4GB RAM and 64-bit Windows 7 operating system. The parameters for CPLEX solver are set to its default values, except that the maximum computation time is set to 3600s and the MIPGap is set to 10^−12^.

Tables 4 and 5 show the CPLEX results of the homogeneous fleet and heterogeneous fleet instances respectively. The columns N, D and T provide the number of buses required, the deadhead bus travel distance, and the CPU time that are used by the CPLEX solver. The deadhead bus travel distance is defined as the idle distance covered by the bus between the destination school of the first trip to the first bus stop of the next trip without carrying any students, when a bus can serve the two trips in sequence. The column Gap indicates the CPLEX solution status: optimal or the optimality gap. The instances of homo1~homo60 can be solved with optimal solutions; while the instances of homo70~homo100 can be solved with suboptimal solutions. Since the new version of CPLEX is used in our research, we get better solutions with the less number of buses for large instances of homo70~homo100. For the last two instances (homo90 and homo100), we get longer travel distances. However, we provide the solutions with less buses, which will reduce the total cost of school bus service. Note that our optimal solution for homo60 is different from that in literature [25].

**Table 4:** CPLEX results for the homogeneous fleet instances.

Instance	MIPHO[25]				MIPHO-B			
	N	D	T(s)	Gap(%)	N	D	T(s)	Gap(%)
homo1	3	0	0.33	opt.	3	0	0.02	opt.
homo2	10	119	0.06	opt.	10	119	0.06	opt.
homo3	10	418	0.08	opt.	10	418	0.05	opt.
homo4	10	441	0.08	opt.	10	441	0.03	opt.
homo5	15	380	0.08	opt.	15	380	0.03	opt.
homo10	17	806	0.17	opt.	17	806	0.09	opt.
homo20	45	1265	0.38	opt.	45	1265	0.19	opt.
homo30	57	2132	25.72	opt.	57	2132	7.88	opt.
homo40	63	3091	91.31	opt.	63	3091	7.86	opt.
homo50	88	3421	241.31	opt.	88	3421	22.75	opt.
homo60	90	4260	1303.55	opt.	90	4255	192.16	opt.
homo70	101	4962	1 hour	-	99	4921	1 hour	1.01
homo80	109	5296	1 hour	-	106	5252	1 hour	5.43
homo90	124	5212	1 hour	-	117	5864	1 hour	11.23
homo100	135	5425	1 hour	-	127	6211	1 hour	16.52

**Table 5:** CPLEX results for the heterogeneous fleet instances.

Instance	MIPHE[25]				MIPHE-B			
	N	D	T(s)	Gap(%)	N	D	T(s)	Gap(%)
hetero1	7	0	0.015	opt.	7(1,2,4)	0	0.02	opt.
hetero2	7	231	0.078	opt.	7(2,2,3)	231	0.03	opt.
hetero3	9	377	0.235	opt.	9(2,3,4)	377	0.06	opt.
hetero4	11	359	0.281	opt.	11(1,3,7)	359	0.05	opt.
hetero5	17	402	1.266	opt.	17(5,4,8)	412	0.06	opt.
hetero10	24	924	7.703	opt.	24(6,5,13)	963	0.19	opt.
hetero20	**-**	**-**	**-**	**-**	50(14,14,22)	1476	0.36	opt.
hetero30	**-**	**-**	**-**	**-**	56(11,17,28)	2917	52.98	opt.
hetero40	**-**	**-**	**-**	**-**	67(16,15,36)	3301	209.68	opt.
hetero50	**-**	**-**	**-**	**-**	76(16,20,40)	3471	803.81	opt.
hetero60	**-**	**-**	**-**	**-**	88(17,22,49)	4205	1 hour	2.3
hetero70	**-**	**-**	**-**	**-**	104(17,23,64)	5392	1 hour	4.5
hetero80	**-**	**-**	**-**	**-**	119(26,28,65)	5474	1 hour	4.1
hetero90	**-**	**-**	**-**	**-**	132(28,25,79)	5774	1 hour	23.8
hetero100	**-**	**-**	**-**	**-**	146(32,28,86)	5899	1 hour	26.4

Table 5 also shows the number of each type of buses used for the instances. The numbers in bracket denote the number of buses with capacity of 40, 50 and 70 respectively. The CPLEX results show that our model MIPHE-B for the heterogeneous fleet school bus scheduling problem outperforms the model MIPHE significantly. Since the number of buses is rather large compared to the types of buses, the bus-based formulation of MIPHE is computationally more complex than the bus type-based formulation. Thus, the CPLEX can only solve the small instances (hetero1~hetero10). Our bus type-based formulation MIPHE-B reduces the model complexity in terms of the number of decision variables, the number of constraints, and the model file size. Modeling the instances with MIPHE-B, CPLEX can solve larger instances (hetero20~hetero50) optimally; the optimal problem size is increased from 58 (hetero10) trips to 286 trips (hetero50). For the large instances (hetero60~hetero100), the CPLEX can provide feasible solutions, and the results for some instances (hetero60~hetero80) are near to the optimal solutions. At the same time, since the difference between the fixed costs of buses is not considered in MIPHE model, the optimal solutions for a instance obtained from MIPHE and MIPHE-B may be different and cannot be compared directly. For example, the bus travel distances for hetero5 and hetero10 from MIPHE-B are longer than that from MIPHE. However, we provide the optimal configuration of the three types of buses. The parameter of fixed bus cost (*F*_*k*_) in MIPHE-B model can guide the MIP solver to select smaller, lower cost buses as many as possible.

The heuristic results for HOMO and HETERO instances are listed in Tables 6 and 7 respectively. Since simulated annealing is a generic probabilistic metaheuristic, the solver proposed in Section 3 was executed 10 times over each instance. For both homogeneous fleet and heterogeneous fleet instances, the size of neighborhood list (*NList*) is set to *n*/2, where *n* is the number of bus trips. The initial temperature for simulated annealing is set to 5.0/*n*, and the cooling rate α is 0.995. The maximum number of loops (*maxloop*) is set to 200. The best, average and standard deviation of the number of buses and the total distance among the 10 solutions are listed in columns N_best_, N_ave_, N_dev_ or D_best_, D_ave_, D_dev_, which are useful to verify the effectiveness of the algorithm.

**Table 6:** Heuristic results for the homogeneous fleet instances.

Instance	SA-heuristic									Heuristic[25]	
	N_best_	N_ave_	N_dev_	N_gap_(%)	D_best_	D_ave_	D_dev_	D_gap_(%)	T(s)	N	D
homo1	3	3	0	0.00	0	0	0	0.00	0.22	3	0
homo2	10	10	0	0.00	119	119	0	0.00	0.33	10	119
homo3	10	10	0	0.00	418	418	0	0.00	0.66	10	418
homo4	10	10	0	0.00	441	441	0	0.00	0.81	10	441
homo5	15	15	0	0.00	380	380	0	0.00	1.23	15	394
homo10	17	17	0	0.00	806	806	0	0.00	3.35	17	838
homo20	45	45	0	0.00	1283	1286.7	7.27	1.40	22.26	45	1393
homo30	58	58	0	1.72	2018	2026.6	7.31	-5.65	64.81	57	2445
homo40	63	63	0	0.00	3124	3140.2	12.73	1.06	147.48	65	3320
homo50	88	88	0	0.00	3457	3489.8	34.32	1.04	509.31	88	3757
homo60	90	91	0.47	0.00	4873	4307.9	52.54	12.68	829.48	94	4266
homo70	103	103.5	0.53	3.88	4590	4545.6	77.29	-7.21	1841.86	106	4565
homo80	108	109.2	0.63	1.85	4798	4686.6	70.92	-9.46	1503.25	111	5023
homo90	116	116.1	0.32	-0.86	4890	4902.7	32.74	-19.92	3071.60	117	5466
homo100	124	124.8	0.63	-2.42	5357	5304.3	59.01	-15.94	3460.68	126	5902
Average	57.3	57.6	0.2	0.53	2436.9	2390.3	23.6	-5.65	730.2	58.3	2556.5

**Table 7:** Heuristic results for the heterogeneous fleet instances.

Instance	N_best_	N_ave_	N_dev_	D_best_	D_ave_	D_dev_	T(s)	Gap1	Gap2	Gap3
hetero1	7(1,2,4)	7	0	0	0	0	0.58	0.00	0.00	0.00
hetero2	7(2,2,3)	7	0	231	231	0	0.61	0.00	0.00	0.00
hetero3	9(2,3,4)	9	0	377	377	0	0.66	0.00	0.00	0.00
hetero4	11(1,3,7)	11	0	359	359	0	0.75	0.00	0.00	0.00
hetero5	17(5,4,8)	17	0	412	419.2	2.5	1.11	0.00	0.00	0.00
hetero10	24(6,5,13)	24	0	978	988.6	6.6	2.93	0.00	1.53	0.00
hetero20	50(14,14,22)	50	0	1535	1557.7	10.2	10.69	0.00	3.84	0.00
hetero30	57(11,18,28)	59	0.94	2785	2678.8	72.0	74.69	1.75	-4.74	2.04
hetero40	68(13,19,36)	69.4	0.70	3129	3179.3	50.6	112.10	1.47	-5.50	2.26
hetero50	77(15,22,40)	78.3	0.67	3607	3531.1	62.7	246.75	1.30	3.77	1.67
hetero60	89(16,24,49)	90.1	0.74	4293	4244.2	41.2	571.29	1.12	2.05	1.42
hetero70	103(16,27,60)	104	0.82	4844	4836.6	73.9	966.03	-0.97	-11.31	-1.42
hetero80	117(25,30,62)	117.2	0.42	5141	5265.9	55.6	1081.67	-1.71	-6.48	-2.25
hetero90	117(25,29,63)	117.9	0.74	5517	5557.8	93.8	1806.31	-12.82	-4.66	-16.61
hetero100	126(23,34,69)	126.4	0.52	5958	5991.7	39.3	2034.31	-15.87	0.99	-18.88

Table 6 shows the heuristic results for the homogeneous fleet instances. The columns N_best_, N_ave_ and N_dev_ represent the best, average and standard deviation of the number of buses among the 10 solutions respectively. The columns D_best_, D_ave_ and D_dev_ represent the best, average and standard deviation of the distance among the 10 solutions respectively, and the column N_gap_ and D_gap_ represents the percentage difference of the number of buses and distance between the best heuristic solution and the CPLEX solution for each instance in Table 4, respectively. The column T denotes the solver time in seconds.

Compared with CPLEX, the heuristic approach to homogeneous fleet school bus scheduling can find the optimal solutions for small-size instances (homo1~homo10), sub-optimal solutions for medium-size instances (homo20~homo60), and better solutions for large-size instances (homo90 and homo100). In addition, compared with the solution results in literature [25], our SA-heuristic algorithm outperforms Kim’s heuristic algorithm in terms of the number of buses and the total travel distance; for large instances homo60~homo100, on average, the number of buses and the total travel distance are reduced by 2.35% and 2.83% respectively.

**Fig 2:**
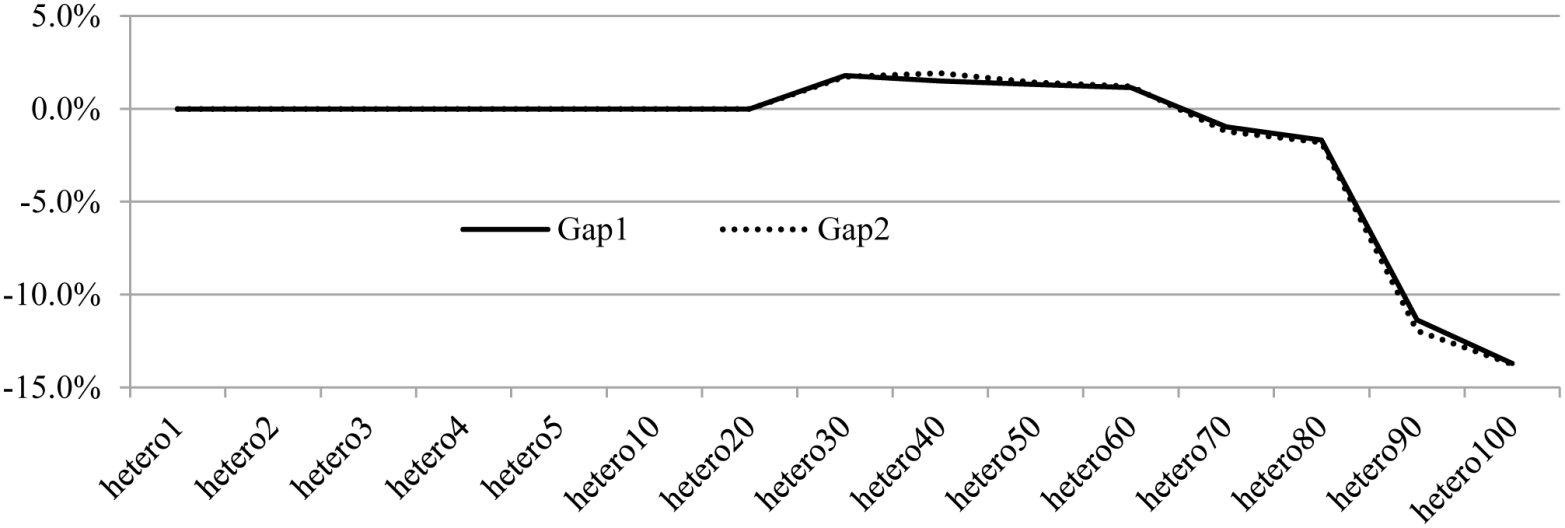
Objective difference between metaheuristic and CPLEX solutions for the heterogeneous fleet instances.

Table 7 shows the heuristic results for the heterogeneous fleet instances. Compared with the solutions in literature[25], we provide the number of buses by bus type. In addition, the objective differences between the heuristic solutions and CPLEX solutions are shown in column Gap1, Gap2 and Gap3, which denote the percentage gaps of the number of buses, the fixed cost of buses and the travel distance respectively. The solution comparison between exact and heuristic approach shown in Fig 2 is interesting. For small-size instances (hetero1~hetero5, the number of trips between 7 and 32), both the two approaches can provide optimal solutions in terms of the number of buses, the total fixed cost and the total travel distance. For medium-size instances (hetero10~hetero50, the number of trips between 58 and 286), CPLEX can find the optimal solutions, and our algorithm can find good enough results. For example, our algorithm can find the optimal number of buses on instances hetero10 and hetero20. For large-size instances (hetero60~hetero100, the number of trips between 342 and 562), CPLEX is able to find sub-optimal solution with small objective gaps for instance hetero60~hetero80, whereas high quality solutions for instances hetero90 and hetero100 are difficult to find. However, the heuristic approach has obvious advantage in solving the larger instances in both the solution quality and computation time.

### 4.2. Results for RSRB and CSCB instances

Another set of sixteen benchmark instances are selected from literature [27]. The instances shown in Table 8 are classified into two groups: the random spatial distribution of schools and bus stops (RSRB) and the clustered distribution (CSCB). These instances have different numbers of students, bus stops and schools. The capacity of the vehicle is assumed to be 66, and its average speed is 20 miles per hour. For the purpose of comparison, the service time of picking up or delivering students at each stop is calculated by the regression model developed in [20], the same as in literature [27]. The maximum riding time (MRT) of students in a bus is set to 2700 seconds (45 minutes) or 5400 seconds (90 minutes). Since there is no bus depot information in the benchmark instances, we assume the bus depot is located at coordinates (105600, 105600).

**Table 8:** The school bus routing and scheduling instances RSRB and CSCB.

Instance	#schools	#stops	#students	#trips1	#trips2
RSRB01	6	250	3409	60	55
RSRB02	12	250	3670	76	62
RSRB03	12	500	6794	131	112
RSRB04	25	500	6805	158	120
RSRB05	25	1000	13765	251	225
RSRB06	50	1000	12201	293	215
RSRB07	50	2000	26912	486	437
RSRB08	100	2000	31939	658	542
CSCB01	6	250	3907	69	64
CSCB02	12	250	3204	76	57
CSCB03	12	500	6813	152	112
CSCB04	25	500	7541	193	128
CSCB05	25	1000	16996	332	277
CSCB06	50	1000	18232	410	309
CSCB07	50	2000	27594	614	449
CSCB08	100	2000	27945	745	484

For school bus scheduling, the bus trips for each school are solved by the metaheuristic algorithm proposed in Section 3. The travel distance *d*_*ij*_ between any two nodes is a float number in inches and is calculated by Manhattan distance. The travel time *t*_*ij*_ between any two nodes is an integer number in seconds, it is estimated by the travel distance and the average bus speed, i.e.*t*_*ij*_ *d*_*ij*_/29.333333. The pickup time at student stop is an integer number estimated by the formula 19+2.6*n (*n* is the number of students at the stop); and the delivery service time at school stop is also an integer number, which is estimated by the formula 29+1.9*n (*n* is the number of students served by the trip). The output trip data includes these features: the trip ID, the first student stop ID and its coordinates, the destination school ID and its coordinates and time windows, the bus travel distance, the trip service time, and the number of students served by the trip. The service time for each trip includes the bus travel time, the students’ pickup time and unloading time at the destination school. The column #trips1 in Table 8 presents the total number of trips for each instance when the MRT is 2700 seconds; and column #trips2 presents the total number of trips for each instance when the MRT is 5400 seconds.

Based on the trips for each school, we build all the models for the instances according to the MIPHO-B formulations proposed in Section 2.2. The parameter *M* in objective function is set to 10^9^. Parameters such as bus travel distance and time are also calculated, same as the settings in single-school problem. The models are solved by CPLEX 12.6. The CPLEX parameters are set to default values, except that the maximum computation time is set to 3600s and the MIPGap is set to 10^−15^.

All the instances in Table 8 are also solved by our solver for homogeneous fleet school bus scheduling problem. The solver was executed 10 times over each instance. The initial temperature for simulated annealing is set to 1.2/*n*, where *n* is the number of bus trips of each instance. The cooling rate α is set to 0.99. The size of neighborhood list (*NList*) is set to *n*/2; and the maximum number of loops (*maxloop*) is 200.

Tables 9 and 10 exhibit the computational results for RSRB and CSCB instances respectively. The 2700 and 5400 in column Instance denote the students’ maxmium riding time in seconds. The columns N, D and T denote the total number of buses, the total bus travel distance and the solver time in seconds. The column Gap indicates the solution status for CPLEX solver; the columns N_gap_ and D_gap_ refer to the percentage difference of the number of buses and the travel distance between CPLEX solutions and the heuristic solutions. For the metaheuristic solver, the columns N_best_, N_dev_, D_best_, D_dev_ have the same meaning as the corresponding columns in Table 6.

**Table 9:** Results for the RSRB benchmark instances.

Instance	CPLEX				SA-Heuristic						
	N	D	Gap(%)	T(s)	N_best_	N_dev_	N_gap_	D_best_	D_dev_	D_gap_	T(s)
RSRB01_2700	26	6914083	opt.	0.06	26	0.00	0.00	6914092.46	0.00	0.00	2.80
RSRB02_2700	27	10028708	opt.	0.09	27	0.00	0.00	10120784.23	0.00	0.92	4.18
RSRB03_2700	48	15761527	opt.	0.42	48	0.00	0.00	15761542.48	14616.9	0.00	19.86
RSRB04_2700	58	16209789	opt.	0.49	58	0.00	0.00	16284162.52	0.00	0.46	25.28
RSRB05_2700	94	26414196	opt.	1.44	94	0.00	0.00	26414264.91	23555.3	0.00	109.58
RSRB06_2700	87	25484524	opt.	1.76	87	0.00	0.00	25572260.46	68555.4	0.34	253.93
RSRB07_2700	149	42328760	opt.	9.92	149	0.00	0.00	42509078.51	29725.2	0.43	1014.68
RSRB08_2700	136	46767310	opt.	1785.92	139	0.52	2.21	46560688.64	175338.4	0.44	1652.50
RSRB01_5400	27	6304173	opt.	0.05	27	0.00	0.00	6304182.28	1361.2	0.00	2.79
RSRB02_5400	23	8665826	opt.	0.08	23	0.00	0.00	8665846.47	0.00	0.00	2.67
RSRB03_5400	46	12812623	opt.	0.2	46	0.00	0.00	12819179.52	12392.1	0.05	11.37
RSRB04_5400	41	11084739	opt.	0.37	41	0.00	0.00	11084771.23	59225.6	0.00	13.63
RSRB05_5400	80	22208728	opt.	1.22	80	0.00	0.00	22264731.97	0.00	0.25	54.66
RSRB06_5400	72	19492477	opt.	1.11	72	0.00	0.00	19541829.61	60773.4	0.25	42.66
RSRB07_5400	141	38837810	opt.	5.94	141	0.00	0.00	38916850.74	45393.8	0.20	543.31
RSRB08_5400	139	42579350	opt.	312.00	140	0.67	0.72	42749715.12	141358.9	0.40	1305.45
Average	74.63	21993413.9	-	132.57	74.88	0.07	0.18	22030248.82	39518.5	0.18	316.21

**Table 10:** Results for the CSCB benchmark instances.

Instance	CPLEX				SA-Heuristic						
	N	D	Gap(%)	T(s)	N_best_	N_dev_	N_gap_	D_best_	D_dev_	D_gap_	T(s)
CSCB01_2700	32	8238295	opt.	0.06	32	0.00	0.00	8238320.96	0.00	0.00	4.03
CSCB02_2700	26	9047666	opt.	0.19	26	0.00	0.00	9049453.10	5413.7	0.02	4.09
CSCB03_2700	58	15180749	opt.	0.62	58	0.00	0.00	15180775.18	0.00	0.00	23.81
CSCB04_2700	62	20111639	opt.	0.58	62	0.00	0.00	20111678.69	14866.7	0.00	37.91
CSCB05_2700	113	29009570	opt.	17.18	114	0.00	0.88	29373744.55	0.00	1.26	191.49
CSCB06_2700	120	31034650	opt.	13.43	120	0.00	0.00	31153106.99	33607.9	0.38	432.20
CSCB07_2700	173	44803980	opt.	41.09	173	0.00	0.00	45124978.25	175032.9	0.72	1413.69
CSCB08_2700	149	51386250	opt.	1767.13	154	0.92	3.36	51352702.16	273384.7	0.07	3349.67
CSCB01_5400	29	7429868	opt.	0.11	29	0.00	0.00	7429893.53	0.0	0.00	3.45
CSCB02_5400	22	6934233	opt.	0.09	22	0.00	0.00	6934243.00	0.0	0.00	2.32
CSCB03_5400	42	10125785	opt.	0.19	42	0.00	0.00	10125802.50	495.0	0.00	10.50
CSCB04_5400	45	13752831	opt.	0.2	45	0.29	0.00	13752859.94	95146.0	0.00	17.55
CSCB05_5400	99	23805471	opt.	9.19	100	0.00	1.01	24087785.53	0.00	1.19	98.58
CSCB06_5400	104	23525340	opt.	3.46	104	0.00	0.00	23552967.39	23467.6	0.12	172.86
CSCB07_5400	146	32561350	opt.	10.83	146	0.00	0.00	32620032.80	2473.6	0.18	445.88
CSCB08_5400	130	37933090	opt.	46.97	130	0.63	0.00	38707240.90	162863.8	2.04	758.36
Average	84.38	22805047.9	-	119.46	84.81	0.12	0.33	22924724.09	49172.0	0.36	435.40

There are several findings from Tables 9 and 10. First, the number of buses can be reduced significantly by bus scheduling. Comparing to the single-school based bus route plan, only about 30% of buses are needed for both RSRB and CSCB instances. Second, the homogeneous fleet bus scheduling problem, which is modeled as VRPTW without capacity constraints, can be efficiently solved by CPLEX with optimal solutions. Third, the local search algorithm coupling with simulated annealing is also effective to solve homogeneous fleet bus scheduling problem. The algorithm proposed in this research can also find the minimum number of buses for the benchmark instances except a few instances.

Table 11 shows the comparison of our algorithm with the heuristic algorithm in [27]. Z_Park_ and Z_SA_ denote the number of required buses obtained from the two algorithms. The Gap column shows the percentage improvement of our algorithm, which is computed as (Z_Park_-Z_SA_)/Z_Park_. On average, our route solutions reduce the number of required buses by 24.8% and 15.98% for RSRB and CSCB instances respectively. It is mainly due to Park’s algorithm is a construction heuristic algorithm with simple improvement operators only.

**Table 11:** Comparison of our algorithm with existing algorithm.

Instances	Z_Park_[27]	Z_SA_	Gap(%)	Instances	Z_Park_[27]	Z_SA_	Gap(%)
RSRB01_2700	35	26	34.62	CSCB01_2700	39	32	17.95
RSRB02_2700	32	27	18.52	CSCB02_2700	33	26	21.21
RSRB03_2700	66	48	37.50	CSCB03_2700	66	58	12.12
RSRB04_2700	68	58	17.24	CSCB04_2700	72	62	13.89
RSRB05_2700	124	94	31.91	CSCB05_2700	135	114	15.56
RSRB06_2700	103	87	18.39	CSCB06_2700	138	120	13.04
RSRB07_2700	185	149	24.16	CSCB07_2700	201	173	13.93
RSRB08_2700	178	139	28.06	CSCB08_2700	183	154	15.85
RSRB01_5400	31	27	14.81	CSCB01_5400	35	29	17.14
RSRB02_5400	30	23	30.43	CSCB02_5400	27	22	18.52
RSRB03_5400	61	46	32.61	CSCB03_5400	52	42	19.23
RSRB04_5400	56	41	36.59	CSCB04_5400	57	45	21.05
RSRB05_5400	106	80	32.50	CSCB05_5400	115	100	13.04
RSRB06_5400	82	72	13.89	CSCB06_5400	123	104	15.45
RSRB07_5400	159	141	12.77	CSCB07_5400	164	146	10.98
RSRB08_5400	158	140	12.86	CSCB08_5400	156	130	16.67
Average	92.13	74.89	24.80	Average	99.75	84.81	15.98

## Conclusions

For a multi-school system, the school bus scheduling problem seeks to optimize bus schedules to serve all the given trips considering the school time windows. Two approaches to solve the bi-objective school bus scheduling problem are discussed in this paper. This research manages to make two contributions: the rewriting of MIP formulation for heterogeneous-fleet school bus scheduling, and the design of a local search algorithm coupling with simulated annealing to minimize the number of buses and the total travel distance.

This research also reports three findings. First, the school bus scheduling problem can be efficiently solved with optimal or sub-optimal solution by MIP solver CPLEX. School bus scheduling problems is a class of combinatorial optimization problem, and is also NP-complete problem. However, along with the great progress in integer programming, especially the introduction of hybrid heuristics in MIP solver design, it is possible to solve large-size real-world school bus scheduling problems by MIP optimizer. For homogenous fleet problem, the CSCB and RSRB instances with up to 745 trips and the HOMO instances with up to 372 trips are solved optimally. For heterogeneous fleet problem, the HETERO instances with up to 286 trips can be solved optimally.

Second, the bus type-based integer programming formulation for heterogeneous fleet problem is more useful in solving real-world problems. Such formulation can reduce the model complexity in terms of the number of decision variables, the number of constraints, and the size of model file. Our benchmark test shows the optimally solvable problem size is increased from 58 trips to 286 trips. At the same time, the fixed costs for each type of buses are considered in the model formulation, which will guide the solver to select as many low priced buses as possible.

Third, we propose a general local search algorithm coupling with simulated annealing to minimize the number of buses and total travel distance. The VRP local search operators, such as one-point move, two-point move, 2-opt move and cross-exchange, are used to improve the route solution gradually. Based on this algorithm, the two-stage metaheuristic solvers for bi-objective school bus routing problem or school bus scheduling problem are designed. The test on the RSRB and CSCB instances shows that our algorithm outperforms the existing mixed load heuristic significantly. Compared to the optimal solutions obtained by CPLEX, our algorithm can also provide the optimal numbers of buses for most of the RSRB and CSCB instances. The test on HETERO instances shows that our metaheuristic approach is of obvious advantages for large-size heterogeneous fleet problem in terms of the solution quality and computation efficiency. In addition, the algorithm can be easily integrated with GIS for solving real-world school bus routing and scheduling problem.

For school bus scheduling problem, this paper and most of existing literature focus on the problems with fixed school time windows. However, the benefit of bus scheduling depends largely on the settings of school bell times. For a multi-school system, if the bell times are adjusted properly, the solution for school bus routing and scheduling problems can be improved substantially. Future works might have considered the problems of school bus scheduling and bell time adjustment simultaneously.

## Supporting Information

S1 File The benchmark instances files of school bus scheduling problem(SBSP). (RAR)

S2 File The benchmark instances files of school bus routing problem(SBRP). (RAR)

S3 File The single school route solution files for SBRP benchmark instances. (RAR)
