# Correction: Exact and Metaheuristic Approaches for a Bi-Objective School Bus Scheduling Problem

**DOI:** 10.1371/journal.pone.0153614

**Published:** 2016-04-07

**Authors:** Xiaopan Chen, Yunfeng Kong, Lanxue Dang, Yane Hou, Xinyue Ye

There is an error in the last sentence of the eighth paragraph of the “4.1. Results for HOMO and HETERO instances” subsection of the Computational Results section. The correct sentence is: In addition, compared with the solution results in literature [25], our SA-heuristic algorithm outperforms Kim’s heuristic algorithm in terms of the number of buses and the total travel distance; for large instances homo60~homo100, on average, the number of buses and the total travel distance are reduced by 2.35% and 7.84% respectively.

In Table 6, there are errors in columns D_best_ and D_gap_(%) in rows homo60, homo70, homo80, homo90, homo100 and Average. In Table 7 there are errors in columns D_best_ and Gap2 in rows hetero30, hetero50, hetero60, hetero70, hetero90, hetero100. Please see the corrected Tables 6 and 7 here.

**Table 6:** Heuristic results for the homogeneous fleet instances.

Instance	SA-heuristic									Heuristic[25]	
	N_best_	N_ave_	N_dev_	N_gap_(%)	D_best_	D_ave_	D_dev_	D_gap_(%)	T(s)	N	D
homo1	3	3	0	0.00	0	0	0	0.00	0.22	3	0
homo2	10	10	0	0.00	119	119	0	0.00	0.33	10	119
homo3	10	10	0	0.00	418	418	0	0.00	0.66	10	418
homo4	10	10	0	0.00	441	441	0	0.00	0.81	10	441
homo5	15	15	0	0.00	380	380	0	0.00	1.23	15	394
homo10	17	17	0	0.00	806	806	0	0.00	3.35	17	838
homo20	45	45	0	0.00	1283	1286.7	7.27	1.40	22.26	45	1393
homo30	58	58	0	1.72	2018	2026.6	7.31	-5.65	64.81	57	2445
homo40	63	63	0	0.00	3124	3140.2	12.73	1.06	147.48	65	3320
homo50	88	88	0	0.00	3457	3489.8	34.32	1.04	509.31	88	3757
homo60	90	91	0.47	0.00	4227	4307.9	52.54	-0.66	829.48	94	4266
homo70	103	103.5	0.53	3.88	4419	4545.6	77.29	-11.36	1841.86	106	4565
homo80	108	109.2	0.63	1.85	4587	4686.6	70.92	-14.50	1503.25	111	5023
homo90	116	116.1	0.32	-0.86	4830	4902.7	32.74	-21.41	3071.60	117	5466
homo100	124	124.8	0.63	-2.42	5181	5304.3	59.01	-19.88	3460.68	126	5902
Average	57.3	57.6	0.2	0.53	2352.7	2390.3	23.60	-4.66	730.20	58.3	2556.5

**Table 7:** Heuristic results for the heterogeneous fleet instances.

Instance	N_best_	N_ave_	N_dev_	D_best_	D_ave_	D_dev_	T(s)	Gap1	Gap2	Gap3
hetero1	7(1,2,4)	7	0	0	0	0	0.58	0.00	0.00	0.00
hetero2	7(2,2,3)	7	0	231	231	0	0.61	0.00	0.00	0.00
hetero3	9(2,3,4)	9	0	377	377	0	0.66	0.00	0.00	0.00
hetero4	11(1,3,7)	11	0	359	359	0	0.75	0.00	0.00	0.00
hetero5	17(5,4,8)	17	0	412	419.2	2.5	1.11	0.00	0.00	0.00
hetero10	24(6,5,13)	24	0	978	988.6	6.6	2.93	0.00	1.53	0.00
hetero20	50(14,14,22)	50	0	1535	1557.7	10.2	10.69	0.00	3.84	0.00
hetero30	57(11,18,28)	59	0.94	2598	2678.8	72.0	74.69	1.75	-12.28	2.04
hetero40	68(13,19,36)	69.4	0.70	3129	3179.3	50.6	112.10	1.47	-5.50	2.26
hetero50	77(15,22,40)	78.3	0.67	3419	3531.1	62.7	246.75	1.30	-1.52	1.67
hetero60	89(16,24,49)	90.1	0.74	4161	4244.2	41.2	571.29	1.12	-1.06	1.42
hetero70	103(16,27,60)	104	0.82	4688	4836.6	73.9	966.03	-0.97	-15.02	-1.42
hetero80	117(25,30,62)	117.2	0.42	5141	5265.9	55.6	1081.67	-1.71	-6.48	-2.25
hetero90	117(25,29,63)	117.9	0.74	5415	5557.8	93.8	1806.31	-12.82	-6.63	-16.61
hetero100	126(23,34,69)	126.4	0.52	5925	5991.7	39.3	2034.31	-15.87	0.44	-18.88
